# ID 276 - Custo-Efetividade do Sulfato de Gentamicina Combinado à Doxiciclina para o Tratamento de Brucelose Humana

**DOI:** 10.5327/2237-9622.2025.v34s1.276

**Published:** 2025-11-25

**Authors:** Endi Lanza Galvão, Sarah Nascimento Silva, Gláucia Cota

**Keywords:** avaliação de tecnologias em saúde, brucelose humana, análise de custo-efetividade

## Abstract

**Introdução:**

A brucelose é uma doença negligenciada, reemergente, causada por bactérias do gênero sp. Trata-se da doença bacteriana mais prevalente no mundo. No Brasil, o manejo da brucelose humana é cercado por muitas incertezas diante do grande contingente de casos assintomáticos, subnotificação e desafios no tratamento da doença, o que motivou a elaboração de diretriz clínica para elaborar recomendações baseadas em evidências. O objetivo do trabalho foi avaliar a eficácia e a segurança da combinação de sulfato de gentamicina e doxiciclina (GT + DX) no tratamento da brucelose humana, comparada com a combinação de sulfato de estreptomicina e doxiciclina (STP + DX).

**Método:**

Para identificar a efetividade das intervenções, realizou-se busca sistemática por ensaios clínicos randomizados nas bases PubMed, EMBASE, Cochrane Library e LILACS, indexados até 28 de setembro de 2023. A mensuração dos custos dos medicamentos utilizou os esquemas posológicos recomendados pela literatura científica e estimativas de preços praticados no Banco de Preços em Saúde. Uma análise de custo-efetividade foi realizada utilizando modelagem por árvore de decisão, usando a cura clínica como desfecho de interesse. Realizaram-se análises de sensibilidade univariadas e probabilísticas.

**Resultados:**

Dois ensaios clínicos randomizados foram identificados na literatura, os resultados comparativos não apontaram diferenças nas taxas de sucesso terapêutico entre a combinação GT + DX versus STP + DX (RR: 0,58, IC 95%: 0,29 a 1,14). As razões de custo-efetividade para GT + DX e STP + DX foram R$ 50,82 e R$ 77,21, respectivamente. A razão de custo-efetividade incremental (ICER – do inglês, ) foi de R$ 535,09/por cura. A análise de sensibilidade univariada indicou que a incerteza quanto aos custos do esquema STP + DX foi o parâmetro com maior impacto no ICER. A análise probabilística corrobora os resultados iniciais, mostrando que GT + DX apresenta um incremento no custo total de tratamento e benefício clínico levemente superior.

**Conclusão:**

A combinação GT + DX, apesar de apresentar um pequeno incremento de custo no tratamento, representa uma alternativa potencialmente custo-efetiva no longo prazo, dependendo do contexto local de preços e da disponibilidade de medicamentos no âmbito do programa estratégico da brucelose humana. Os resultados do estudo podem influenciar as políticas de saúde no que se refere à escolha de regimes antibióticos para o tratamento da brucelose, especialmente em áreas endêmicas. A incorporação de GT + DX deve ser considerada pelos formuladores de políticas de saúde, levando em conta a relação custo-efetividade e a acessibilidade do tratamento.

